# Supplementary material for: Functional Domains of the Herpes Simplex Virus Type 1 Tegument Protein pUL37: The Amino Terminus is Dispensable for Virus Replication in Tissue Culture
Source: Viruses. 2019 Sep 14;11(9):853. doi: 10.3390/v11090853 (PMC6783895; doi:10.3390/v11090853)
Supplement: Supplementary file 1 [file viruses-11-00853-s001.pdf]

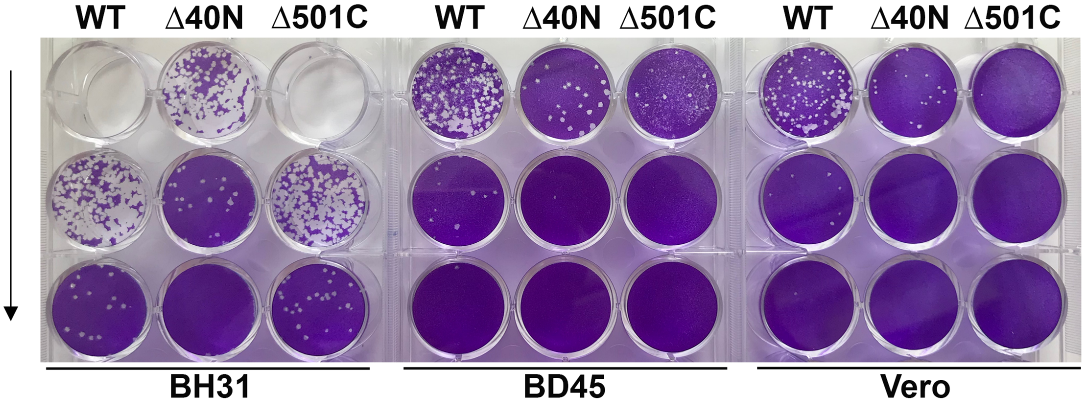

Supplementary Figure S1. Plating efficiency of mutant viruses following marker-rescue/marker-transfer transfections. BH31 (UL36/UL37) cells were co-transfected with KΔ36/37CR and plasmids carrying UL37 wild-type (WT) and UL37-EGFP truncation mutations, Δ40N, and Δ501C. The transfection progeny was plated on BH31, BD45 (UL37) and Vero cells to demonstrate marker-rescue/marker-transfer. The transfection progeny contained mostly KΔ36/37CR virus compared to the recombinant virus, at least 100 fold more. WT and Δ40N were able to plaque on all cells whereas Δ501C only replicated on the complementing cell lines. The arrow indicates increasing 10-fold serial dilutions down the plate starting at a 1×10^{-2} dilution of the transfection virus stock.

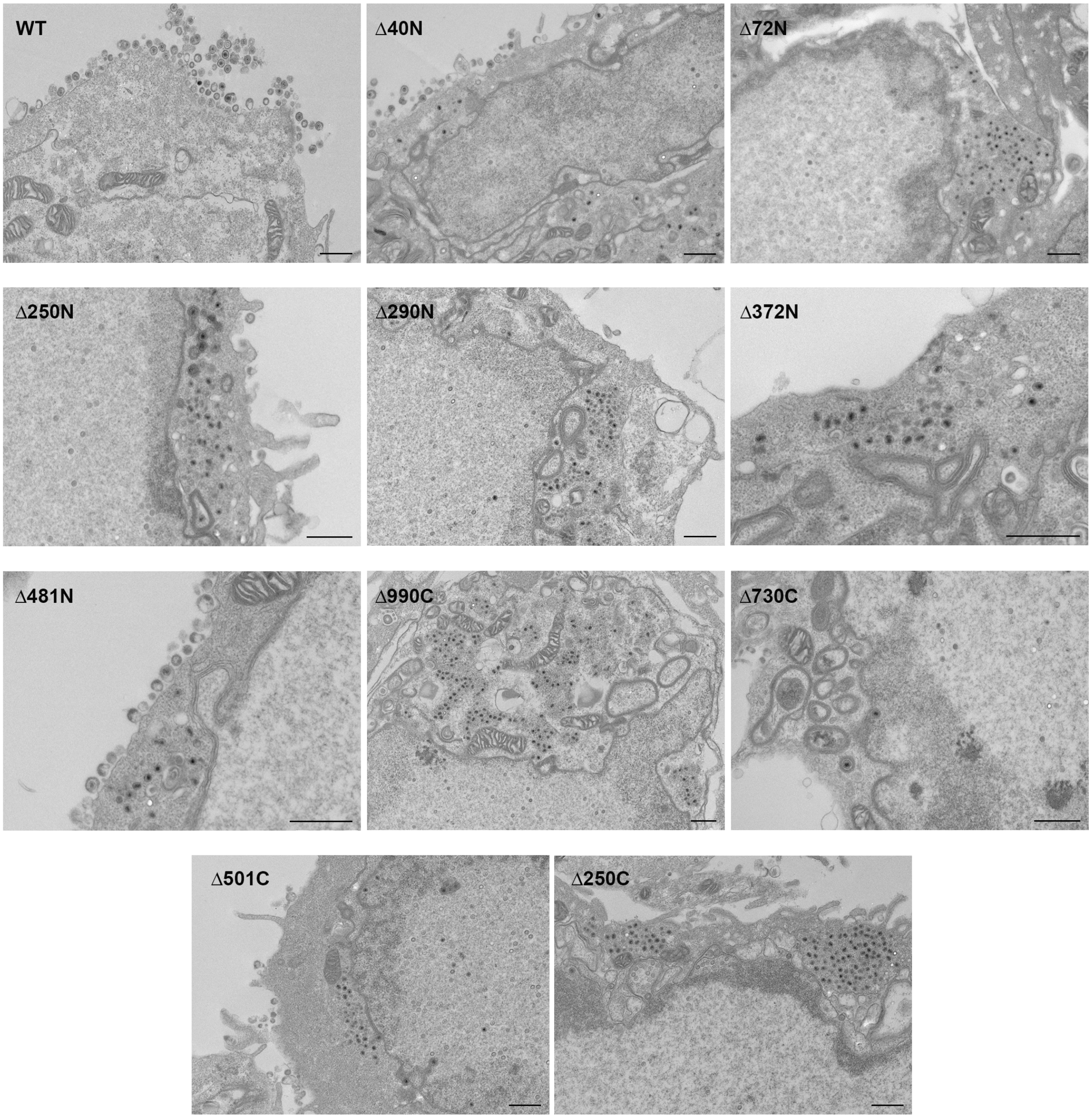

Supplementary Figure S2. Truncations of pUL37 lead to a defect in cytoplasmic envelopment of virus particles. Representative TEM images of cells infected with each virus encoding a polypeptide truncation mutant of pUL37 are shown with scale bars of 1 μm indicated.

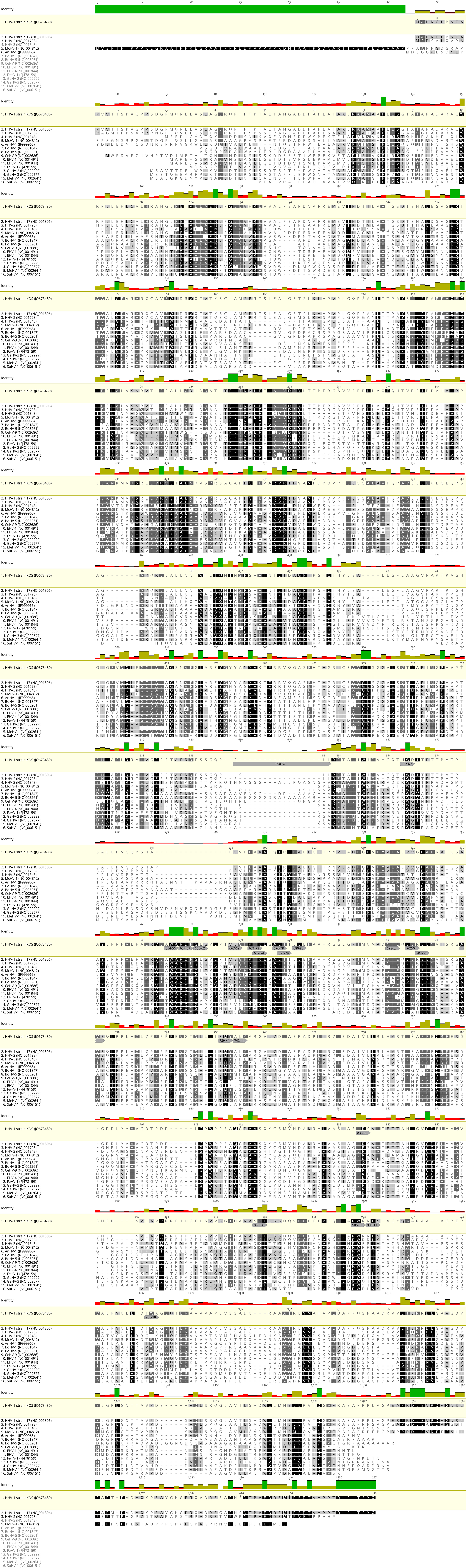
Identity
1. HHV-1 strain KOS (JQ673480)
2. HHV-1 strain 17 (NC_001806)
3. HHV-2 (NC_001798)
4. HHV-3 (NC_001348)
5. McHV-1 (NC_004812)
6. AnHV-1 (JF999965)
7. BoHV-1 (NC_001847)
8. BoHV-5 (NC_005261)
9. CeHV-9 (NC_002686)
10. EHV-1 (NC_001491)
11. EHV-4 (NC_001844)
12. FeHV-1 (FJ478159)
13. GaHV-2 (NC_002229)
14. GaHV-3 (NC_002577)
15. MeHV-1 (NC_002641)
16. SuHV-1 (NC_006151)

Supplementary Figure S3. pUL37 alanine-scanning mutants (AAA) target conserved residues in the C-terminal half of the polypeptide. pUL37 amino acid sequences or homologs from different alphaherpesviruses were aligned using Geneious and amino acid conservation is indicated. pUL37 AAA mutated sequences are annotated on KOS pUL37 (Accession: JQ673480).

Supplementary Table S1. Primer sequences.

Primer name	Nucleotide sequence 5′-3′
BglII F	GAGCCGGGCGTC**ATG***AGATCT*GCAGACCGCGGTCTC
BglII R	GAGACCGCGGTCTGC*AGATCT*CATGACGCCCGGCTC
BsrGI^{s}F	GGAATTCTACGTAAAAACTTTCAGGCG*TGTACA*GGGCGCCAGCGAGCACAC
BsrGI^{s}R	GTGTGCTCGCTGGCGCCC*TGTACA*CGCCTGAAAGTTTTTACGTAGAATTCC
WT BglII F	GGA*AGATCT*ATGGCAGACCGCGGTCTCCCGTCC
Δ40 BglII F	GGG*AGATCT*CCAACCCCCACGGCCGAGACGGC
Δ72 BglII F	GGG*AGATCT*GGAACGGCCATCGCCCCGGCAGAC
Δ250N BglII F	GGG*AGATCT*GCGCACCTCCAGCGCATAGACGAC
Δ290 BglII F	GGG*AGATCT*CTGTTGGCGCAGTTTCAGCACACC
Δ372 BglII F	GGG*AGATCT*GCCGTCTCCAGCCTGCTGCAGCTC
Δ481 BglII F	GGG*AGATCT*GTAAAAACTTTCAGGCGGGTCCAG
WT BsrGI R	GTGCTCGCTGGCGCCC*TGTACA*CG
Δ990 SpeI R	CC*ACTAGT*GTCGCGACGGCTCGTAAGCACGAC
Δ730 SpeI R	GG*ACTAGT*CTCGGGGGGCGGGGGGGGCTGCAG
Δ501 SpeI R	GG*ACTAGT*CGCCTCGCAGAGCCGCCCGTGCGTG
Δ250C SpeI R	GG*ACTAGT*CGCCGAGAACAGCGTCACCCGATTAC
WT SpeI R	GG*ACTAGT*TAACGGCAAGTCCGTGGGTGG
CMV-DsRed F	GG*ACTAGT*TAGTTATTAATAGTAATCAATTAC
CMV-DsRed R	*GGCGGCCGC*AACCACAACTAGAATGCAGTG
550–52 F	AGTTCCGGTCAACCCGCGGCCGCCCGCGAGACGGCGCTC
654–56 F	GCCCTGCGTGTGTTGGCGGCCGCCCGGGACTTTGGGCTG
657–59 F	GTGTTGGCCTGGGCCGCGGCCGCTGGGCTGGGCTATCTC
667–69 F	GGCTATCTCCCCACGGCGGCCGCCCATCGCACCAAACTG
672–74 F	GTTGAGGGCCATCGCGCGGCCGCGGGCGCGCTGATCACC
676–78 F	CGCACCAAACTGGGCGCGGCCGCCACCCTCCTCGAACCG
680–82 F	GGCGCGCTGATCACCGCGGCCGCACCGGCCGCCCGGGGC
702–04 F	GACAACATAGAGCAGGCGGCCGCGGAGCTGTACGTGATC
704–06 F	ATAGAGCAGCTGCTCGCGGCCGCGTACGTGATCTCCAGG
713–15 F	GTGATCTCCAGGGGTGCGGCCGCGCAGCTGCGGCCCCTG
739-41 F	AGCCTCCTGTTGATTGCGGCCGCCGCCCTGGCCGCCCGG
906–08 F	GGTCAGTTTCTAGCGGCGGCCGCGCGTCTGTCGGCCTGC
909–11 F	CTAGCGCGCTGGCGGGCGGCCGCGGCCTGCTATCAAGCC
